# Risk factors for hospital-acquired antimicrobial-resistant infection caused by *Acinetobacter baumannii*

**DOI:** 10.1186/s13756-015-0083-2

**Published:** 2015-10-09

**Authors:** Darcy Ellis, Bevin Cohen, Jianfang Liu, Elaine Larson

**Affiliations:** Mailman School of Public Health, Columbia, University Medical Center, 722 West 168th Street, New York, NY 10032 USA; School of Nursing, Columbia University Medical Center, 630 West 168th Street, New York, NY 10032 USA

## Abstract

**Background:**

*Acinetobacter baumannii* can cause serious healthcare-associated infections (HAIs) and the incidence is increasing, with many strains now resistant to multiple antibiotic classes. The aims of this study were to examine factors associated with HAIs caused by antimicrobial-resistant as compared with antimicrobial-susceptible strains of *A. baumannii* and to investigate trends in the incidence of resistance over time. Electronic data from two U.S. hospitals in a large urban healthcare system in over the years 2006–2012 were used for the analysis. Multiple logistic regression was used to explore risk factors for infection with *A. baumannii* resistant to ampicillin or ampicillin/sulbactam in the bloodstream, urinary tract, and respiratory tract. The Cochran-Armitage trend test was used to explore resistance trends over time.

**Findings:**

A total of 671 adults with first-time *A. baumannii* infection were included in the analysis; 302 isolates (45 %) were resistant to ampicillin or ampicillin/sulbactam and 369 (55 %) were susceptible. In the multivariable analysis, significant risk factors included longer length of stay prior to infection (Odds Ratio [OR] = 1.03; 95 % Confidence Interval [CI]: 1.01, 1.04), hospital A versus B (OR = 0.35; 95 % CI: 0.13, 0.93), and antibiotic use prior to infection (OR = 2.88; 95 % CI: 1.02, 8.13). Resistance was more common in respiratory infections (OR = 2.96; 95 % CI: 1.04, 8.44). No trend was found between year of infection and resistance.

**Conclusions:**

The risk factors we identified are consistent with previous findings, but we found no evidence in this population that resistance to ampicillin or ampicillin/sulbactam was increasing over time.

## Background

*Acinetobacter baumannii* is increasingly implicated as a cause of healthcare-associated infections (HAI), which confer a high risk of morbidity and mortality to patients [1–5]. Infections caused by *A. baumannii* may also be highly resistant to antimicrobials, particularly those strains isolated from critically ill patients in intensive care settings [6–9]. *A. baumannii* isolates that are resistant to antibiotics can worsen outcomes for patients due to delays in administration of effective therapy, limited treatment options, and high toxicity of available therapies [1]. Risk factors for multidrug-resistant *A. baumannii* colonization and infection include prolonged length of hospital stay, exposure to the intensive care unit (ICU), mechanical ventilation, central venous catheterization, urinary catheterization, prior exposure to antimicrobials, greater severity of illness, surgery, and receipt of invasive procedures [1, 10, 11]. Although risk factors for antibiotic-resistant *A. baumannii* infection have been explored in many patient populations, fewer studies have assessed potential differences in risk factors for those infected with antimicrobial-resistant versus susceptible strains.

Antimicrobial resistance among *A. baumannii* appears to be on the rise internationally [8, 12], though temporal changes in antimicrobial susceptibility have not been widely described and may vary locally. Furthermore, research suggests seasonal variation in *A. baumannii* incidence [13], yet research into this association is limited. Therefore, the aims of this study were to examine factors associated with HAIs caused by antimicrobial-resistant as compared with antimicrobial-susceptible strains of *A. baumanni*i, as well as to investigate trends in the incidence of resistance over time.

## Materials and methods

### Study population

This retrospective cohort study was conducted as part of a federally funded project, “Health Information Technology to Reduce Healthcare-Associated Infection” (National Institute of Nursing Research, National Institutes of Health; R01NR010822),” which established a clinical research database of patients hospitalized within a large urban healthcare system in New York City. This analysis included all adult (≥18 years) discharges occurring from 2006 to 2012 in two tertiary/quaternary care hospitals within the system: a 647-bed adult facility and a 914-bed pediatric/adult facility.

### Data collection

The study database was constructed using electronic data from clinical and administrative systems shared between the study institutions [14]. The primary outcome of interest was the first hospital-acquired (i.e., occurring >2 days after hospital admission) bloodstream infection (BSI), urinary tract infection (UTI), surgical site infection (SSI), or pneumonia caused by *A. baumannii*. Infections were identified using a combination of time-stamped microbiology laboratory records and International Classification of Diseases, Ninth Revision, Clinical Modification (ICD-9-CM) procedure and diagnosis codes, based on modified criteria from the Centers for Disease Control and Prevention National Healthcare Safety Network (NHSN) [15]. BSI were defined as positive *A. baumannii* blood culture with no positive culture at another body site within the previous 14 days. UTI were defined as positive *A. baumannii* urine culture, i.e., ≥10^5^ colony forming units (CFU) per mL of urine and no more than one other species of microorganism, or 10^3^-10^5^ CFU/mL plus pyuria. SSI were defined as positive *A. baumannii* wound culture within 30 days of an ICD-9-CM-documented NHSN operative procedure. Pneumonia was defined as positive *A. baumannii* respiratory culture plus any ICD-9-CM code for bacterial pneumonia. Resistance to ampicillin or ampicillin/sulbactam was determined for each infection based on antibiogram data, which were stored electronically for all cultures. The year and season of each infection (Winter, January-March; Spring, April-June; Summer, July-September; Fall, October-December) were also recorded.

Patients’ demographic characteristics and medical conditions were collected from electronic sources. The institution’s electronic medication administration record was used to determine whether patients received antibiotics or high risk medications (including chemotherapeutic agents, immunosuppressants, and anti-inflammatory drugs) at least 24 h before infection. Comorbidities (diabetes, renal failure, and malignancies) and the Charlson Comorbidity Index [16] were collected using ICD-9-CM codes. Electronic administrative records were used to determine patients’ age, sex, length of hospitalization, ICU admission, prior within-network hospitalization, and admission from a skilled nursing facility.

### Statistical analysis

The frequency of BSI, UTI, SSI, and pneumonia caused by antibiotic sensitive and resistant *A. baumannii* were recorded by year. The Cochran-Armitage test for trend was used to assess whether the proportion of *A. baumannii* infections that were resistant to antibiotics changed over time. To explore risk factors for HAI caused by antimicrobial resistant versus susceptible *A. baumannii* strains, bivariate analyses between infection and possible predictors were conducted using Pearson’s *χ*^2^ test for independence (categorical variables) or simple logistic regression (continuous variables). Variables examined included age, gender, hospital (A or B), ICU stay prior to infection, Charlson Comorbidity Index, diabetes, renal failure, malignancy, length of hospital stay prior to infection, antibiotic and high-risk medication use prior to infection, season and year (2006–2012), prior stay in a skilled nursing facility, prior in-network hospitalization, and infection site (BSI, UTI, or pneumonia). SSI were excluded from this analysis due to small sample size. Variables with a p-value less than 0.25 in the bivariate analysis were included in a multivariable logistic regression model in a stepwise forward fashion. All analyses were performed using SAS version 9.4 (SAS Institute, Cary, NC).

## Results

A total of 671 adults with first time hospital-acquired *A. baumannii* infection were identified from 2006 through 2012; 302 patients (45 %) had antimicrobial resistant *A. baumannii* infections and 369 (55 %) had antimicrobial susceptible infections. Differences between patients with antimicrobial resistant versus susceptible *A. baumannii* infections are described in Table 1. In the final multivariable model, the significant predictors of resistance were length of stay prior to infection (OR = 1.03; 95 % CI: 1.01, 1.04), being at hospital A vs. B (OR = 0.35; 95 % CI: 0.13, 0.93), having a respiratory infection versus other infection types (OR = 2.96; 95 % CI: 1.04, 8.44), and antibiotic use prior to infection (OR = 2.88; 95 % CI: 1.02, 8.13).

**Table 1:** Factors associated with healthcare-associated infection caused by antimicrobial resistant versus susceptible *Acinetobacter baumannii* in two New York City hospitals, 2006-2012

	Resistant to ampicillin or ampicillin/sulbactam		
	Yes, *N* (%)	No, *N* (%)	*P*-value*
Total	302 (45 %)	369 (55 %)	
Mean (range) age, in years	61 (19–98)	61 (18–97)	0.65
Sex			
Female	125 (41 %)	160 (43 %)	0.61
Male	177 (59 %)	209 (57 %)	
Hospital			
A	217 (76 %)	190 (58 %)	<0.0001
B	70 (24 %)	139 (42 %)	
ICU prior to infection			
Yes	219 (83 %)	178 (82 %)	0.72
No	44 (17 %)	39 (18 %)	
Mean (range) Charlson Comorbidity Index	5.61 (0–17)	5.39 (0–16)	0.40
Mean (range) days of stay prior to infection	39 (3–377)	18 (3–193)	<0.0001
High risk medication prior to infection			
Yes	136 (72 %)	155 (77 %)	0.24
No	53 (28 %)	46 (23 %)	
Prior stay in skilled nursing facility			
Yes	21 (7 %)	31 (8 %)	0.49
No	281 (93 %)	338 (92 %)	
Prior in-network hospitalization			
Yes	72 (24 %)	74 (20 %)	0.24
No	230 (76 %)	295 (80 %)	
Year of infection onset			
2006	32 (11 %)	69 (19 %)	0.003
2007	67 (22 %)	65 (18 %)	
2008	64 (21 %)	48 (13 %)	
2009	40 (13 %)	52 (14 %)	
2010	36 (12 %)	44 (12 %)	
2011	41 (14 %)	47 (13 %)	
2012	22 (7 %)	44 (12 %)	
Site of infection			
Bloodstream	65 (22 %)	85 (23 %)	0.64
Urinary tract	96 (32 %)	123 (33 %)	0.67
Pneumonia	187 (62 %)	172 (47 %)	<0.0001
Season of infection onset			
Winter	67 (22 %)	83 (22 %)	0.75
Spring	76 (25 %)	95 (26 %)	
Summer	85 (28 %)	113 (31 %)	
Fall	74 (25 %)	78 (21 %)	
Diabetes			
Yes	100 (33 %)	106 (29 %)	0.22
No	202 (67 %)	263 (71 %)	
Renal failure			
Yes	181 (60 %)	179 (49 %)	0.003
No	121 (40 %)	190 (51 %)	
Malignancy			
Yes	69 (23 %)	79 (21 %)	0.65
No	233 (77 %)	290 (79 %)	
Antibiotic use prior to infection			
Yes	16 (40 %)	20 (20 %)	0.017
No	24 (60 %)	78 (80 %)	

Numbers in strata may not equal total due to missing values

*Continuous variables assessed using simple logistic regression (Wald *χ* ^2^). Categorical variables assessed using Pearson’s *χ* ^2^

**Fig. 1:**
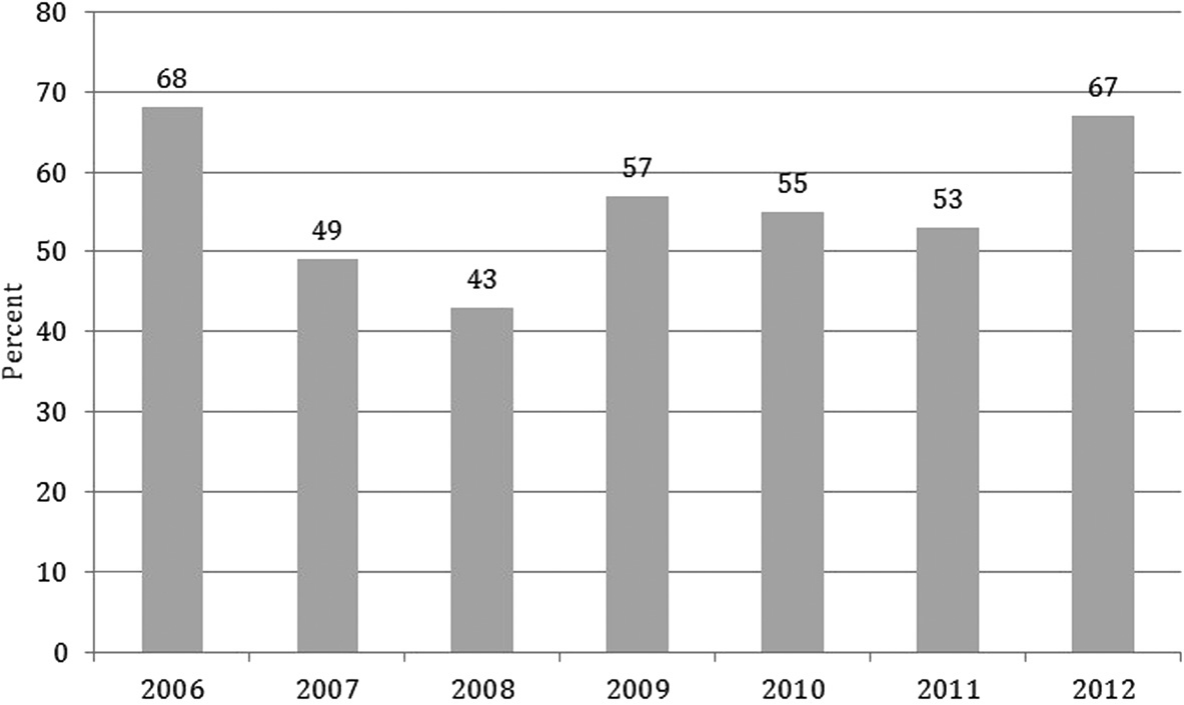
Percent of hospital-acquired *Acinetobacter baumannii* strains resistant to ampicillin or ampicillin/sulbactam in two New York City hospitals, 2006–2012. Pearson’s *χ*2 test for independence across years, *p* = 0.003. Two-sided Cochran-Armitage test for trend, *p* = 0.73

Of the total number of first time *A. baumannii* infections over the study period from 2006 to 2012, 43-68 % were due to strains resistant to antibiotics. Although there were significant differences by year (chi-square test for independence *p* = 0.003), there was no significant trend of increasing or decreasing incidence of resistance over time (Cochran-Armitage *p* = 0.73; Fig. 1).

## Discussion

According to the most recent NHSN report, among *A. baumannii* infections identified in 2009–10, 78 % of catheter-associated urinary tract infections, 67 % of central-line associated bloodstream infections had multidrug resistance, and 99 % of ventilator-associated pneumonia were multidrug resistant [5]. However, other studies have reported rates of multidrug resistance between 51 and 54 % [17, 18], which are closer to the rate of resistance found in this study (45 %). Estimates of the prevalence of resistance may vary due to differences in the definitions of antimicrobial resistance used across studies, as well as differences in the types of infections studied, patient case mix, and geographic location. Seasonality, however, does not appear to play a role.

Although rates of resistance differed by year, there was no significant trend over time. A previous report from New York City comparing resistance to ampicillin/sulbactam across three time points found an increase between 1999 and 2001 (34 % to 46 %), though this trend did not continue into 2006 (44 %) [19]. Other U.S. studies, however, have reported large increases in resistance over the past decade. A study conducted in Detroit found that between 2003 and 2008, the total number of patients with *A. baumannii* infection increased, as did resistance to most antibiotics, with ampicillin/sulbactam resistance climbing from 11 % to 60 % [20]. NHSN described an increase in multidrug resistance covering six classes including ampicillin/sulbactam from 50 % in 2007–8 to 63 % in 2009–10 [5]. It appears, therefore, that there is no consistent trend in ampicillin/sulbactam resistance for *A. baumannii*, and that trends may be primarily associated with local practices or other regional differences.

This study identified four risk factors for hospital-acquired *A. baumannii* resistant infection. Consistent with other studies, a longer hospital stay prior to infection, infection in the respiratory tract, and antibiotic use before infection were all significant predictors of antimicrobial resistance [1, 2, 12]. Greater resistance among respiratory tract infections may be explained in part by high levels of *A. baumannii* contamination on respirators and suctioning equipment, particularly in ICUs, which may lead to environmental reservoirs of resistant strains [21]. This finding highlights the importance of environmental hygiene for preventing *A. baumannii* infections in the respiratory tract, especially for ventilated patients. Unexpectedly, being in hospital A vs. B was also a significant predictor, controlling for potential confounders such as comorbidities, medications used, other host characteristics, and length of stay. There are no obvious reasons why resistance varied significantly between the two hospitals, given that both are tertiary/quaternary care hospitals within the same hospital system. It is possible that there were other host or environmental factors which were unaccounted for, though this could not be assessed due to the retrospective nature of our study. While the infection prevention policies were the same in both hospitals, it is also possible that infection prevention practices varied [22, 23].

There are some limitations to this study. Firstly, we did not examine factors associated with resistance among patients with surgical site infections because the numbers were too few. Secondly, data were limited to what was available from electronic data sources. Although the electronic algorithms used to detect infections are based on NHSN criteria and have been validated by a clinical team, the “gold standard” for identifying infections is clinician diagnosis. In addition, information on the molecular characteristics of *A. baumannii* isolates were not available, so subtyping could not be performed. Furthermore, some patient comorbidities were identified by ICD-9-CM codes, which have been shown to have poor sensitivity for many chronic conditions [24]. Lastly, this study was conducted using data from a single healthcare system in the United States, a region where *A. baumannii* is not endemic. Thus the findings may not be generalizable to other populations.

The results of this study confirm what some other studies have shown, that the length of hospital stay and antibiotic use prior to infection are significantly associated with increased risk of an antimicrobial resistant *A. baumannii* infection, and that resistance was more common in the respiratory tract as compared to other body sites. Unlike other studies investigating risk factors for infection, this study also examined trends in resistance over a seven year period and found no clear pattern in the prevalence of resistance to ampicillin/sulbactam among *A. baumannii* strains.

### Ethics, consent and permissions

This study was approved by the Columbia University Medical Center Institutional Review Board. A waiver of informed consent was granted.
